# Copy number variation (CNV) identification, interpretation, and database from Brazilian patients

**DOI:** 10.1590/1678-4685-GMB-2019-0218

**Published:** 2020-11-13

**Authors:** Victória Cabral Silveira Monteiro de Godoy, Fernanda Teixeira Bellucco, Mileny Colovati, Hélio Rodrigues de Oliveira-Junior, Mariana Moysés-Oliveira, Maria Isabel Melaragno

**Affiliations:** 1 Universidade Federal de São Paulo, Departamento de Morfologia e Genética, Disciplina de Genética, São Paulo, SP, Brazil.

**Keywords:** CNV, copy number variation, database, CNV classification

## Abstract

Copy number variations (CNVs) constitute an important class of variation in the human genome and the interpretation of their pathogenicity considering different frequencies across populations is still a challenge for geneticists. Since the CNV databases are predominantly composed of European and non-admixed individuals, and Brazilian genetic constitution is admixed and ethnically diverse, diagnostic screenings on Brazilian variants are greatly difficulted by the lack of populational references. We analyzed a clinical sample of 268 Brazilian individuals, including patients with neurodevelopment disorders and/or congenital malformations. The pathogenicity of CNVs was classified according to their gene content and overlap with known benign and pathogenic variants. A total of 1,504 autosomal CNVs (1,207 gains and 297 losses) were classified as benign (92.9%), likely benign (1.6%), VUS (2.6%), likely pathogenic (0.2%) and pathogenic (2.7%). Some of the CNVs were recurrent and with frequency increased in our sample, when compared to populational open resources of structural variants: 14q32.33, 22q11.22, 1q21.1, and 1p36.32 gains. Thus, these highly recurrent CNVs classified as likely benign or VUS were considered non-pathogenic in our Brazilian sample. This study shows the relevance of introducing CNV data from diverse cohorts to improve on the interpretation of clinical impact of genomic variations.

## Introduction

CNVs are characterized by losses or gains of DNA sequences that are larger than 50 bp (Alkan *et al.* 2011; MacDonald *et al.* 2014; Zarrei *et al.* 2015). They are a relevant class of variants due to the large number of genome segments that differs in the dosage between individuals, conferring great interindividual diversity (Iafrate *et al.* 2004). Such variants are also present in healthy individuals with no apparent association with disease phenotypes, being considered benign CNVs (Iafrate *et al.* 2004; Sebat *et al.* 2004; Redon *et al.* 2006; Korbel *et al.* 2007; Conrad *et al.* 2010). There are also CNVs that are responsible for the etiology of numerous human diseases, such as multiple syndromes that are associated to congenital anomalies, complex neurodegenerative and neuropsychiatric disorders, intellectual disability, cancer and immunological diseases (Stankiewicz and Lupski 2010; Girirajan *et al.* 2011). The recognition of their influence on the phenotype, however, is not an easy task. Although some CNV maps and databases have been constructed (Zarrei *et al.* 2015) for both, healthy individuals (e.g. DGV- http://dgv.tcag.ca/dgv/app/home) and affected patients (e.g. DECIPHER - https://decipher.sanger.ac.uk/), it is still a great challenge to assess the CNVs’ clinical impact (Lee *et al.* 2007; Gijsbers *et al.* 2011; Brnich *et al.* 2019). The American College of Medical Genetics and Genomics (ACMG) presented guidelines for CNVs interpretation and recommended the use of specific standard terminology: “pathogenic”, “likely pathogenic”, “uncertain significance”, “likely benign”, and “benign” (Richards *et al.* 2015). This guide was recently updated to assist clinical laboratories in the classification and reporting of CNVs. These professional standards will guide the evaluation of constitutional CNVs and encourage consistency and transparency across clinical laboratories (Riggs *et al.* 2019). However, there are no generally established rules for CNV analysis, interpretation, and classification, and the guidelines can change over time due to the scientific information evolution (de Leeuw *et al.* 2012; Palmer *et al.* 2014; Brnich *et al*, 2019; Riggs *et al*. 2019). Furthermore, it has been shown that the CNV distribution can differ across ethnic populations (Li *et al*. 2009; Collins *et al*. 2019). The Brazilian population is highly admixed and still underrepresented in genomic databanks (Naslavsky *et al*. 2017; Andrade *et al*. 2018). Thus, the main goal of this study was to survey and classify large CNVs to assemble a database from Brazilian patients in order to improve the interpretation of their clinical impact.

## Subjects and Methods

### Individuals studied

A sample composed of 268 microarrays performed in patients with phenotypic alterations was studied (Table S1). The patients were recruited from the Medical Genetics Center of the Universidade Federal de São Paulo, outpatient clinics of the Hospital São Paulo and other genetics centers in the state of São Paulo, Brazil. This project was approved by the University Ethics Committee and all participants or parents signed informed consents. All procedures performed involving human participants were in accordance with the ethical standards of the institutional and/or national research committee and with the 1964 Helsinki declaration and its later amendmentsor comparable ethical standards. We studied microarrays from 143 patients with normal karyotypes (69 presented with diverse phenotypic alterations and 74 were patients with phenotype of the oculoauriculovertebral spectrum - OAVS) and 125 patients with previously identified genomic imbalances/chromosomal alterations, who participated in specific studies in our laboratory (50 presented with 22q11.2 deletion, 23 with apparently balanced translocations, nine with marker chromosomes, five with 18q deletions, four with 18p deletions, four with ring chromosomes, and 30 with other abnormal G-banding karyotypes exclusive to a single patient). 

### Microarray-based copy number variation assay and quality control

Genomic DNA was obtained from peripheral blood using the Gentra Puregene kit (Qiagen-Sciences, Maryland, USA). DNA samples were then analyzed using the Genome-Wide Human Array 6.0 SNP array (n= 59 individuals), CytoScan 750K (n= 54 individuals), and CytoScan High-Density SNP array (n= 155 individuals), following the manufacturer’s instructions (Affymetrix, Santa Clara, CA, US). Array analyses were performed using the Chromosome Analysis Suite software (ChAS), version 3.3 (Affymetrix, Santa Clara, CA, USA). The quality control (QC) parameters were applied according to the manufacturer´s recommendations. For Genome-Wide Human Array 6.0 SNP array platform, samples with Median of the Absolute values of all Pairwise Differences (MAPD) ≤ 0.35 were included in the sample. For CytoScan High-Density (HD) SNP array and CytoScan 750K platforms, samples with MAPD ≤ 0.25, SNP quality control (SNPQC) ≥ 15 (or ≥ 12 when all other parameters met the requirements), and waviness standard deviation (waviness SD) ≤ 0.12 (when all other parameters met the requirements) were also included in the sample. 

### CNVs analysis and classification

**Figure 1 -:**
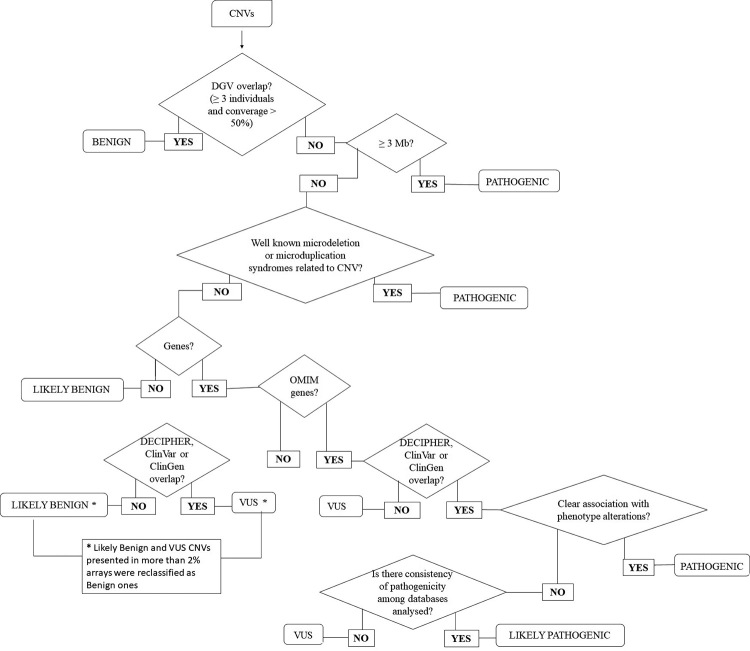
Flowchart for CNVs analysis and interpretation.

The CNV classification was performed by the same investigator in a blind manner considering array type and patients´ phenotype. Autosomal CNVs that had a minimum coverage of 50 probes and a minimum size of 200 kb for gains and 150 kb for losses were considered for the analysis of pathogenicity, since deletions can be more deleterious for the phenotype. The CNVs previously detected by other cytogenomic tests and undoubtedly causative to the patients’ phenotype such as, deletions 18p, 18q and deletion 22q11.2, were excluded from the analysis in order to avoid super representation of these loci. The genomic imbalances were annotated based on the GRCh37/hg19 Genome Build (Feb 2009). CNVs analysis was performed using the UCSC with DGV track (May 15 2016 version), DECIPHER track (Oct 30 2018 version), ClinVar track (Oct 2017 version), ClinGen track (Oct 2017 version), OMIM track for analysis of genes associated with diseases (Oct 10 2018 version) and NCBI RefSeq Genes track (April 19 2017 version). A flowchart for CNVs classification (Figure 1) was built based on the criteria described by Lee *et al*. (2007), Vermeesch *et al*. (2012), Palmer *et al*. (2014), Richards *et al*. (2015), and Nowakowska (2017). CNVs were classified into five categories proposed by the ACMG guidelines (Richards *et al*. 2015): benign, likely benign, variant of uncertain significance (VUS), likely pathogenic and pathogenic. A CNV was considered benign when there was more than 50% overlap, in size and location, with DGV-CNVs of the same nature (i.e., deletion or duplication) from at least three unaffected individuals, and the nonoverlapping segment did not exceed 50 percent of the DGV-CNVs’ length. CNVs were considered likely benign when they did not contain genes or if they did, the genes within them were not OMIM genes and the CNVs did not overlap with the ones found in databases of affected individuals (DECIPHER, ClinVar and ClinGen). CNVs were considered VUS when (1) they contained genes that were not OMIM genes, and they overlapped at least one CNV found in databases of affected individuals, with more than 50% overlap, in size and location; (2) when they contained OMIM genes but did not overlap CNVs found in databases of affected individuals; or (3) when they had OMIM genes and overlapped CNVs found in databases of affected individuals, but do not show a clear correlation with phenotypic alterations and consistency of classifications in the analyzed databases. CNVs were considered likely pathogenic when they presented genes described in OMIM, overlapped CNVs present in databases of genomic imbalances in affected individuals, and had no clear association with phenotypic alterations, but with consistency in the databases indicating phenotypic alterations. CNVs were considered pathogenic when (1) they were more than 3 Mb in length; (2) they overlapped with regions associated with DECIPHER microdeletion/microduplication syndromes; or (3) when, even though their size were not more than 3 Mb in length, they harbored OMIM genes, overlapped with CNVs found in databases of affected individuals that showed consistent correlation with phenotypic alterations**.** After this classification, recurrent CNVs found in a high percentage (≥ 2%) of patients in our sample were reclassified as benign ones. 

## Results

### CNVs type, size and pathogenicity

A total of 1,504 autosome CNVs from 268 microarrays were considered for downstream analysis of pathogenicity. Among them, 1,207 (80.3%) were gains, and 297 (19.7%) were losses. Table 1 present the number and percentage of loss, gain, and total CNVs according to the different sizes and classifications of pathogenicity. According to the classification criteria, 1,397 of them (92.9%) were considered benign, 25 (1.6%) were likely benign, 39 (2.6%) were VUS, 3 (0.2%) were likely pathogenic, and 40 (2.7%) were pathogenic. The mean size of the CNVs was ~763 kb, being ~704 kb for gains and ~1.0 Mb for losses. The mean size of benign, likely benign, VUS, likely pathogenic and pathogenic CNVs was 586 kb, 522 kb, 621 kb, 1.0 Mb and 7.2 Mb, respectively. The sizes of the CNVs ranged from 200 kb to 24.5 Mb for gains and from 150 kb to 20 Mb for losses. The mean number of CNVs per patient was 5.6, being 12.6 for 6.0 SNP array, 2.9 for 750K array, and 3.9 for HD array. 

**Table 1 -:** Number and frequency (in percentage) of CNVs in our sample according to their type of genomic imbalance, size, and classification.

				Losses (%)					Gains (%)					Total (%)				
	<0.5 Mb	0.5 - 1 Mb	1-3 Mb	3-5 Mb	> 5 Mb	Total	<0.5 Mb	0.5 - 1 Mb	1-3 Mb	3-5 Mb	> 5 Mb	Total	<0.5 Mb	0.5 - 1 Mb	1-3 Mb	3-5 Mb	> 5 Mb	Total
B	223 (91.8%)	13 (86.6%)	8 (50%)	0 (0%)	0 (0%)	244 (82.1%)	651 (96.1%)	361 (97.3%)	129 (92.1%)	11 (84.6%)	1 (16.7%)	1153 (95.5%)	874 (95%)	374 (96.9%)	137 (87.8%)	11 (55%)	1 (4.5%)	1397 (92.9%)
LB	12 (4.9%)	0 (0%)	1 (6.3%)	0 (0%)	0 (0%)	13 (4.4%)	8 (1.2%)	1 (0.3%)	3 (2.1%)	0 (0%)	0 (0%)	12 (1%)	20 (2.2%)	1 (0.3%)	4 (2.6%)	0 (0%)	0 (0%)	25 (1.6%)
VUS	6 (2.5%)	0 (0%)	4 (25%)	0 (0%)	0 (0%)	10 (3.4%)	18 (2.7%)	9 (2.4%)	2 (1.5%)	0 (0%)	0 (0%)	29 (2.4%)	24 (2.6%)	9 (2.2%)	6 (3.8%)	0 (0%)	0 (0%)	39 (2.6%)
LP	0 (0%)	1 (6.7%)	1 (6.3%)	0 (0%)	0 (0%)	2 (0.7%)	0 (0%)	0 (0%)	1 (0.7%)	0 (0%)	0 (0%)	1 (0.1%)	0 (0%)	1 (0.3%)	2 (1.3%)	0 (0%)	0 (0%)	3 (0.2%)
P	2 (0.8%)	1 (6.7%)	2 (12.4%)	7 (100%)	16 (100%)	28 (9.4%)	0 (0%)	0 (0%)	5 (3.6%)	2 (15.4%)	5 (83.3%)	12 (1%)	2 (0.2%)	1 (0.3%)	7 (4.5%)	9 (45%)	21 (95.5%)	40 (2.7%)
T	243 (100%)	15 (100%)	16 (100%)	7 (100%)	16 (100%)	297 (100%)	677 (100%)	371 (100%)	140 (100%)	13 (100%)	6 (100%)	1207 (100%)	920 (100%)	386 (100%)	156 (100%)	20 (100%)	22 (100%)	1504 (100%)

P: Pathogenic; LP: Likely pathogenic; VUS: variants of unknown significance; LB: Likely Benign; B: Benign; T: Total.

### Recurrent CNVs in our sample

Benign, likely benign, and VUS CNVs that were found in, arbitrarily, more than 2% arrays within a certain genomic region, were grouped and were considered as recurrent CNVs in our sample. According to these criteria, our sample presented with recurrent copy gains in four genomic regions: 14q32.33 (97.8% patients), 22q11.22 (32.1%), 1p36.32 (9.7%), and 1q21.1 (6,7%). In our pathogenicity evaluation, these first two copy gains had been classified as benign, the third as VUS and the fourth as likely benign. Given their high frequency, VUS CNVs in 1p36.32 and likely benign CNVs in 1q21.1 were reclassified as benign (i.e. non-pathogenic) CNVs in our Brazilian sample and already included in analyzes (Table S2).

## Discussion

In our array sample, a higher number of gain CNVs (80.3%) compared to loss CNVs (19.7%) was found, in agreement with previous studies from the literature (Kang *et al*. 2008; Pietiläinen *et al*. 2011; Palmer *et al*. 2014). Most of the CNVs in the present study were classified as benign (92.9%), and about half (62.6%) of those were smaller than 500 kb. Furthermore, in accordance to the literature (Lee *et al*. 2007), we observed that the CNV mean size increased according to pathogenicity, that is, the greater the imbalance, the greater the pathogenicity. We found that the CNVs distribution differed between platforms. Although the 6.0 SNP array platform was used in only 22% of the performed arrays, 49.6% of the CNVs were identified in this platform, with a mean of 12.6 CNVs/individual, while 750K and HD platforms showed averages of 2.9 and 3.9 CNVs/individual, respectively, that indicate a significant difference (p<0.001). The mean number of CNVs per patient was 5.6, being 12.6 for Human Array 6.0 SNP array, 2.9 for 750K array and 3.9 for HD array. These differences could be related to the characteristics of the arrays used. The 6.0 SNP array platform presents more than 1.8 million probes distributed along all the genome including probes in segmental duplication regions and in pseudogenes, while the 750 K and HD arrays, containing about 750,000 and 2.7 million probes, respectively, are more recent technologies than the 6.0 SNP array platform and are composed of probes with greater specificity and sensitivity, focusing in clinically relevant regions that results in a higher accuracy in genomic imbalances detection. It is important to highlight that this difference between platforms is not associated with QC metrics, since only high-quality genotyping reactions were considered in this analysis. Thus, we must consider that the variation of the array type, company origin, and the filtering used for CNV analysis, are factors that may difficult the comparison between published datasets and the CNVs reported in open databases. Some benign, VUS and likely benign CNVs were found to be recurrent in our sample and showed high frequency in four genomic regions (14q32.33, 22q11.22, 1p36.32, and 1q21.1), being exclusively gain CNVs. The 14q32.33 and 22q11.22 CNVs, found in all three different platforms, were classified as benign in all individuals due to overlap with CNVs from the DGV. In contrast, some 1q21.1 and 1p36.32 CNVs were reclassified considering their high frequency in our sample (Table S2). The 1p36.32 CNVs were found exclusively in 750k (35.2% patients) and HD (4.5%) platforms and were reclassified as benign CNVs due to their high frequency. Interestingly, among the seven CNVs found using the HD platform, five were first classified as benign by DGV, one was classified first as VUS and after reclassified as benign due their high frequency, and another was maintained as likely benign because it had a different genomic coordinates (chr1:2412626-2729513) comparing to the others.

The 1q21.1 gain CNVs were found only in the 6.0 SNP array platform (20.3% patients) and HD (3.9%) platforms. Among the 12 CNVs found in the 6.0 SNP array platform, only one was first classified as benign by DGV while the other 11 were further reclassified as benign ones. In the HD platform, the smaller five out the six CNVs found were classified as benign, and the other, larger (952kb), was reclassified as benign. It is important to note that, among the reclassified CNVs, all of them were larger than the CNV classified first as benign or presented little different coordinates. Thus, although these findings reveal differences in CNV distribution between platforms, there was a consistency in recurrent CNVs within each platform, which demonstrates the reliability of our classification criteria.

We looked for these recurrent CNVs in other studies from the literature. Benign 14q32.33 gain CNV was also found in a high frequency (>90%) in some studies from populations with European, African or East Asian ancestry (de Smith *et al*. 2007; Conrad *et al*. 2010) but not in other studies in individuals from Ontario, Thailand and Caucasians and African-Americans (Sebat *et al*. 2004; Shaikh *et al*. 2009; Suktitipat *et al*. 2014; Uddin *et al*. 2015). The 22q11.22 gain CNV, considered as benign, had a higher frequency in our sample comparing with studies from DGV that also referred a CNV in this region (Itsara *et al*. 2009; Shaikh *et al*. 2009; Cooper *et al*. 2011). Deletions in the 22q11.2 region have already been described in patients with OAVS (Xu *et al*. 2008; Digilio *et al*. 2009). Given the previous association of this region to the OAVS spectrum, there could result in a bias in the CNV frequency in this region in our sample. However, among the 268 individuals analyzed in the present study, this 22q11.22 CNV was found in the 30 out of 74 (40.5%) patients with a clinical diagnosis of OAVS and in 56 out of 194 (28.9%) patients with other diseases. Thus, this CNV was found not only in OAVs patients in our sample, but also in patients with different phenotypes, indicating that this CNV can be found in a higher frequency in the Brazilian population. The 1q21.1 gain CNV, classified at first as likely benign, was also considered as recurrent in our sample. In the DGV database, this variant was reported in only one study, in which a 1q21.1 gain was observed in two out of 29,084 individuals from the USA and Canada (Coe *et al*. 2014). Due to the high frequency of this CNV in our sample, we reclassified this CNV as benign. Analogously, the 1p36.32 gain CNV, primely considered as VUS, was reclassified as benign due to its recurrence in our sample. This variant has a higher frequency in our sample when compared to cohorts described in DGV that detected this CNV in other ancestries (Redon *et al*. 2006) (the HapMap collection). These differences in CNV frequencies between the reported data from the literature and our data may indicate distinct composition of the individuals studied since there is a great populational heterogeneity among the publications. However, these frequency divergencies may also reflect differences in the ability in CNV detection, since a diversity of approaches (such as ROMA, BAC-aCGHs, and SNP array) are used for gain and loss detection. This is a relevant factor that does not permit a reliable comparison between data obtained from different papers from the literature, which can show variability in the detection of certain CNVs, as shown in our data from three different platform used, even from the same company. The data obtained in our study indicated that the established analysis flowchart was highly effective in the classification of the CNVs’ pathogenicity and allowed the establishment of an CNVs database based on Brazilian individuals. This resource has been remarkably valuable for the diagnostic screenings in our laboratory, considering admixed genetic background of the Brazilian population. The data leveraged in this study may contribute for the pathogenicity interpretation of CNVs in other populations underrepresented in currently available open resources for structural variants.

## Supplementary material

Table S1 - Detailed information about all CNVs analyzed in our sample.

Table S2 - Recurrent gain CNVs in 1p36.32 and 1q21.1 showing their interpretation after re-classification accounting for CNV populational frequency.
