# Supplementary material for: Copy number variation (CNV) identification, interpretation, and database from Brazilian patients
Source: Genet Mol Biol. 2020 Nov 13;43(4):e20190218. doi: 10.1590/1678-4685-GMB-2019-0218 (PMC7783508; doi:10.1590/1678-4685-GMB-2019-0218)
Supplement: Table S2 - [file 1415-4757-GMB-43-4-e20190218-s2.pdf]

Genetics and Molecular Biology

https://doi.org/10.1590/1678-4685-GMB-2019-0218

Supplementary Material to "Copy number variation (CNV) identification, interpretation, and database from Brazilian patients"

Table S2 - Recurrent gain CNVs in 1p36.32 and 1q21.1 showing their interpretation after re-classification accounting for CNV populational frequency

Array Type	Chr	Cytoband Start	Cytoband End	Start	End	Size (kbp)	Type	Segment Interpretation	Justification
					**1p36.32**				
HD array	1	p36.32	p36.32	3421593	3628023	206,43	Gain	Benign	DGV
HD array	1	p36.32	p36.32	3417596	3628023	210,427	Gain	Benign	DGV
HD array	1	p36.32	p36.32	3331773	3544104	212,331	Gain	Benign	DGV
HD array	1	p36.32	p36.32	3397288	3628023	230,735	Gain	Benign	DGV
HD array	1	p36.32	p36.32	2619887	2889789	269,902	Gain	Benign	DGV
HD array	1	p36.32	p36.32	3318713	3628023	309,31	Gain	Benign	Reclassified CNV
HD array	1	p36.32	p36.32	2412626	2729513	316,887	Gain	Likely Benign	No OMIM genes/Not present in clinical databases
750 array	1	p36.32	p36.32	3340960	3627839	286,879	Gain	Benign	Reclassified CNV
750 array	1	p36.32	p36.32	3340960	3627839	286,879	Gain	Benign	Reclassified CNV
750 array	1	p36.32	p36.32	3340960	3627839	286,879	Gain	Benign	Reclassified CNV
750 array	1	p36.32	p36.32	3340960	3627839	286,879	Gain	Benign	Reclassified CNV
750 array	1	p36.32	p36.32	3340960	3627839	286,879	Gain	Benign	Reclassified CNV
750 array	1	p36.32	p36.32	3340854	3627839	286,985	Gain	Benign	Reclassified CNV
750 array	1	p36.32	p36.32	3340783	3627839	287,056	Gain	Benign	Reclassified CNV

Array Type	Chr	Cytoband Start	Cytoband End	Start	End	Size (kbp)	Type	Segment Interpretation	Justification
750 array	1	p36.32	p36.32	3340783	3627839	287,056	Gain	Benign	Reclassified CNV
750 array	1	p36.32	p36.32	3340783	3627839	287,056	Gain	Benign	Reclassified CNV
750 array	1	p36.32	p36.32	3338302	3627839	289,537	Gain	Benign	Reclassified CNV
750 array	1	p36.32	p36.32	3336608	3627839	291,231	Gain	Benign	Reclassified CNV
750 array	1	p36.32	p36.32	3332409	3627839	295,43	Gain	Benign	Reclassified CNV
750 array	1	p36.32	p36.32	3328515	3627839	299,324	Gain	Benign	Reclassified CNV
750 array	1	p36.32	p36.32	3328515	3627839	299,324	Gain	Benign	Reclassified CNV
750 array	1	p36.32	p36.32	3328515	3627839	299,324	Gain	Benign	Reclassified CNV
750 array	1	p36.32	p36.32	3318964	3627839	308,875	Gain	Benign	Reclassified CNV
750 array	1	p36.32	p36.32	3318964	3627839	308,875	Gain	Benign	Reclassified CNV
750 array	1	p36.32	p36.32	3318964	3627839	308,875	Gain	Benign	Reclassified CNV
750 array	1	p36.32	p36.32	3318964	3627839	308,875	Gain	Benign	Reclassified CNV
**1q21.1**									
HD array	1	q21.1	q21.1	145195964	145398202	202,238	Gain	Benign	DGV
HD ARRAY	1	q21.1	q21.1	144279309	144884761	605,452	Gain	Benign	DGV
HD array	1	q21.1	q21.1	144048367	144884970	836,603	Gain	Benign	DGV
HD ARRAY	1	q21.1	q21.1	144048367	144884970	836,603	Gain	Benign	DGV
HD array	1	q21.1	q21.1	143983190	144842529	859,339	Gain	Benign	DGV
HD ARRAY	1	q21.1	q21.1	143932349	144884970	952,621	Gain	Benign	Reclassified CNV
6.0 SNP array	1	q21.1	q21.1	144007037	144876884	869,847	Gain	Benign	Reclassified CNV
6.0 SNP ARRAY	1	q21.1	q21.1	144007037	144876884	869,847	Gain	Benign	Reclassified CNV
6.0 SNP array	1	q21.1	q21.1	144007037	144876884	869,847	Gain	Benign	Reclassified CNV
6.0 SNP ARRAY	1	q21.1	q21.1	144007037	144876884	869,847	Gain	Benign	Reclassified CNV

Array Type	Chr	Cytoband Start	Cytoband End	Start	End	Size (kbp)	Type	Segment Interpretation	Justification
6.0 SNP array	1	q21.1	q21.1	144007037	144876884	869,847	Gain	Benign	Reclassified CNV
6.0 SNP ARRAY	1	q21.1	q21.1	144007037	144876884	869,847	Gain	Benign	Reclassified CNV
6.0 SNP array	1	q21.1	q21.1	144007037	144876884	869,847	Gain	Benign	Reclassified CNV
6.0 SNP ARRAY	1	q21.1	q21.1	144007037	144880405	873,368	Gain	Benign	Reclassified CNV
6.0 SNP array	1	q21.1	q21.1	144007037	144880405	873,368	Gain	Benign	Reclassified CNV
6.0 SNP ARRAY	1	q21.1	q21.1	144007037	144880405	873,368	Gain	Benign	Reclassified CNV
6.0 SNP array	1	q21.1	q21.1	144007037	144880405	873,368	Gain	Benign	Reclassified CNV
6.0 SNP ARRAY	1	q21.1	q21.1	144034084	144916995	882,911	Gain	Benign	DGV
